# Evaluating Threats in Multinational Marine Ecosystems: A Coast Salish First Nations and Tribal Perspective

**DOI:** 10.1371/journal.pone.0144861

**Published:** 2015-12-21

**Authors:** Joseph K. Gaydos, Sofie Thixton, Jamie Donatuto

**Affiliations:** 1 The SeaDoc Society, UC Davis Karen C. Drayer Wildlife Health Center–Orcas Island Office, Eastsound, Washington, United States of America; 2 Swinomish Indian Tribal Community, La Conner, Washington, United States of America; Swedish University of Agricultural Sciences, SWEDEN

## Abstract

Despite the merit of managing natural resources on the scale of ecosystems, evaluating threats and managing risk in ecosystems that span multiple countries or jurisdictions can be challenging. This requires each government involved to consider actions in concert with actions being taken in other countries by co-managing entities. Multiple proposed fossil fuel-related and port development projects in the Salish Sea, a 16,925 km^**2**^ inland sea shared by Washington State (USA), British Columbia (Canada), and Indigenous Coast Salish governments, have the potential to increase marine vessel traffic and negatively impact natural resources. There is no legal mandate or management mechanism requiring a comprehensive review of the potential cumulative impacts of these development activities throughout the Salish Sea and across the international border. This project identifies ongoing and proposed energy-related development projects that will increase marine vessel traffic in the Salish Sea and evaluates the threats each project poses to natural resources important to the Coast Salish. While recognizing that Coast Salish traditions identify all species as important and connected, we used expert elicitation to identify 50 species upon which we could evaluate impact. These species were chosen because Coast Salish depend upon them heavily for harvest revenue or as a staple food source, they were particularly culturally or spiritually significant, or they were historically part of Coast Salish lifeways. We identified six development projects, each of which had three potential impacts (pressures) associated with increased marine vessel traffic: oil spill, vessel noise and vessel strike. Projects varied in their potential for localized impacts (pressures) including shoreline development, harbor oil spill, pipeline spill, coal dust accumulation and nearshore LNG explosion. Based on available published data, impact for each pressure/species interaction was rated as likely, possible or unlikely. Impacts are likely to occur in 23 to 28% of the possible pressure/species scenarios and are possible in another 15 to 28% additional pressure/species interactions. While it is not clear which impacts will be additive, synergistic, or potentially antagonistic, studies that manipulate multiple stressors in marine ecosystems suggest that threats associated with these six projects are likely to have an overall additive or even synergistic interaction and therefore impact species of major cultural importance to the Coast Salish, an important concept that would be lost by merely evaluating each project independently. Failure to address multiple impacts will affect the Coast Salish and the 7 million other people that also depend on this ecosystem. These findings show the value of evaluating multiple threats, and ultimately conducting risk assessments at the scale of ecosystems and highlight the serious need for managers of multinational ecosystems to actively collaborate on evaluating threats, assessing risk, and managing resources.

## Introduction

For decades, scientists, managers and politicians have acknowledged the merit of managing natural resources on the scale of ecosystems [1]. Place-based management, however, can be challenging when ecosystems cross international boundaries. While increasing in popularity, terrestrial multinational "transfrontier" conservation parks in Southern Africa have faced challenges associated with setting priorities and instituting collective action by the multiple countries and governments involved [2]. Similar challenges have been documented in efforts to manage marine ecosystems that span multiple countries (e.g. [3]). Evaluating threats and managing risk in multinational ecosystems requires each government involved to consider actions in concert with other actions being taken by co-managing countries. When the ecosystems being managed are not established parks or do not have some type of oversight group, it is easy for individual parties to overlook cumulative risk at the ecosystem level. This management oversight of not "thinking ecosystem" is occurring in the Salish Sea, a 16,925 km^**2**^ inland sea shared by Washington State (USA), British Columbia (Canada), and Indigenous Coast Salish governments.

The Salish Sea is considered an international treasure. Like many coastal ecosystems around the world, however, it is under significant pressure from a growing human population, the overharvest of many natural resources, changing oceanic and atmospheric conditions, and the conversion of natural habitat to urban development [4]. Despite the ecological understanding that ecosystems benefit from ecosystem-level management rather than from management that stops at political boundaries, there is no active, over-arching mechanism for the local, state, provincial, federal and Coast Salish governments overseeing natural resources in the Salish Sea to collaborate on resource management [4]. Consequently, when governing bodies within the Salish Sea evaluate the costs and benefits of proposed development activities, they fail to take into account other proposed projects occurring outside of their jurisdiction, but within the ecosystem. As a result efforts to evaluate threats, and to ultimately assess risk, are incomplete.

Multiple fossil fuel and port development projects that will increase marine vessel traffic are underway or being considered on the US and Canadian side of the Salish Sea. Each project has the potential to create jobs, improve trade and improve the economic situation in the region. They also have the potential for negative environmental consequences, as the vessel traffic associated with these projects is expected to increase underwater vessel noise, increase risk of vessel collision or vessel strike of wildlife, increase oil spills, increase exposure to coal-associated contaminants in biota, impact access to or availability of watchable wildlife, and greatly impact human access to the harvest and consumption of fish and wildlife. Nearshore development activities associated with these projects also have the potential to negatively impact natural resources. In order to conduct effective planning and decision-making in light of the proposed developments, it is imperative to have an understanding of the range of threats and potential impacts and any additive, synergistic, or antagonistic interactions, on both ecological and human health [5,6]. Despite this, there is no legal mandate or mechanism requiring a comprehensive review of the potential threats and cumulative impacts of these multiple energy-related development activities throughout the Salish Sea and across the international border.

Currently, almost 7 million people reside within the watersheds of this inland sea, and Coast Salish First Nations and Tribes have inhabited the region since time immemorial. Despite modern political divisions, the indigenous Coast Salish have always recognized the Salish Sea as an integral entity in Coast Salish lifeways, with symbiotic interactions between humans and the Salish Sea, and they work collaboratively to view the ecosystem in its entirety, without being hindered by international borders. One example is the Coast Salish Gathering, a platform for Washington State Tribal leaders, British Columbia First Nation Chiefs, and U.S. and Canadian regulatory agencies to meet and work on mutual goals. The Gathering fosters a “policy dialogue” that brings major environmental-related issues to the attention of government officials in a common voice, expressing the many values of the indigenous traditions and knowledge (www.coastsalishgathering.com).

In the United States, Tribes have called for a more comprehensive and cumulative impact assessment methodology that accurately and effectively evaluates how resource-based development projects can impact social, cultural and community lifeways [7–10]. This is because Tribes have been significantly absent from ecological and health risk assessments and risk management as most assessments and management strategies fail to mention the impacts that resource-based development activities can have on tribal communities, tribal homelands, unadjudicated Aboriginal rights, or treaty-guaranteed hunting, fishing, and gathering rights [8,11]. Current risk assessment methods fail to account for the fundamental worldviews and relationships that connect Native peoples with the physical, ecological and spiritual worlds, which form the foundation of health and wellbeing [7,9,10]. Recognizing that the multiple proposed fossil fuel-related and port development projects in the Salish Sea have the potential to negatively impact natural resources that are important to the Coast Salish, and consequently impact health and wellbeing, there is great interest in assessing cumulative impacts of these activities on both sides of the border.

In this project we identify ongoing and proposed energy-related development projects that will increase marine vessel traffic in the Salish Sea, we identify threats associated with them, and we enumerate the potential impact that these threats pose to 50 natural resources important to the Coast Salish, setting the stage for a more comprehensive assessment of cumulative risks.

## Materials and Methods

Considering the deeply held values about symbiotic relationships that the Coast Salish peoples hold between themselves and the natural resources of the Salish Sea [10,12], increased marine vessel traffic in the region has the ability to impact many facets of Coast Salish health and wellbeing. Assessing the many possible impacts are beyond the scope of this report. Instead, this work focuses on how proposed or on-going energy-related port development projects could affect natural resources that are important to the Coast Salish, specifically “culturally important species.”

### Expert Elicitation of Culturally Important Species

Recognizing that Coast Salish traditions identify all species as important and connected, making prioritization challenging, Coast Salish and academics specializing in Coast Salish traditional resource use were asked to provide names of species that are especially important or of major concern. Species or subspecies were included if they met one or more of the following criteria:

1. The species is heavily depended upon for harvest revenue
2. The species is heavily depended upon as a staple food source
3. The species is especially culturally or spiritually significant
4. Historically (even if not currently) the species has been part of Coast Salish lifeways.

The final list of Coast Salish species of major importance was reviewed and recommended by members of the Coast Salish Gathering.

### Identification of on-going or proposed energy-related developments

All known ongoing or proposed energy-related development projects in the Salish Sea that are expected to substantially increase marine vessel traffic were considered. Only those projects that involved vessel traffic and could be verified using site development plans, public scoping documents, or project profiles produced by the developer were included. While some projects, such as the Snohomish County (Washington) Public Utility District proposed tidal energy project (USA Federal Energy Regulatory Commission Project No. 12690–005) were evaluated, they were not included because they did not meet the increased vessel traffic criteria.

### Evaluating impacts to Natural resources

Peer-reviewed data were used to estimate potential for a project component (pressure) to directly harm the species identified through the expert elicitation. Each project was broken down into two gross categories: increased vessel traffic (with subcategories of an oil spill during transit, increased vessel noise, and vessel strike of an animal) and localized impacts (with subcategories of shoreline development, harbor spill, pipeline spill, coal dust accumulation, or explosion as applicable). For each species/pressure component, literature was reviewed to see if the pressure had been documented to have a negative effect on the species. Specifically, searches were conducted for each species and pressure combination. If data were not available for a specific species, additional searches were conducted using closely related species or taxa and that pressure. If data were available demonstrating the pressure had the potential to harm the identified species, the pressure was considered likely to impact that species (Table 1). If it had not been shown to cause damage for that species but had for a closely related species, impact was considered possible (Table 1). When the literature showed no impact, the pressure was considered unlikely to cause impact (Table 1). If data were not available for assessing the species/pressure interaction, the pressure was identified as data deficient (Table 1). For spatially explicit or spatially limited threats (localized impacts such as shoreline development, harbor spill, pipeline spill, coal dust accumulation, or nearshore liquefied natural gas explosion), the habitat range of the species based on its natural history, specifically the animal's propensity to occur in a defined area, was considered for each location. If data and natural history of a species overlaid to demonstrate that a pressure could impact a species, impact was identified as likely. If literature demonstrated a direct effect on a similar species but not on the exact species, and the pressure spatially overlapped with the habitat occurrence of the species, impact was considered possible. If the data did not show supporting potential impact, if literature was found showing no impact, or if a species was known to not occur within the range of the potential pressure, impact was considered unlikely. In cases where lack of data prevented evaluation of impact, the species/pressure component was cited as data deficient. Impacts to identified species via negative effect(s) on indicator prey species were not evaluated. In all cases, the concerns identified here must be evaluated in light of the U.S. Federal Court decisions concerning Treaty Rights of the United States Tribes.

**Table 1:** Impact ranking criteria.

Impact Ranking	Criteria
Likely	Data demonstrates potential impact; species distribution and pressure overlap spatially
Possible	Data demonstrates potential impact to similar species; species distribution and pressure overlap spatially
Unlikely	Data demonstrates no impact; species distribution and pressure do not overlap spatially
Data Deficient	Insufficient data to assess

## Results

### Ongoing or proposed development projects

**Fig 1:**
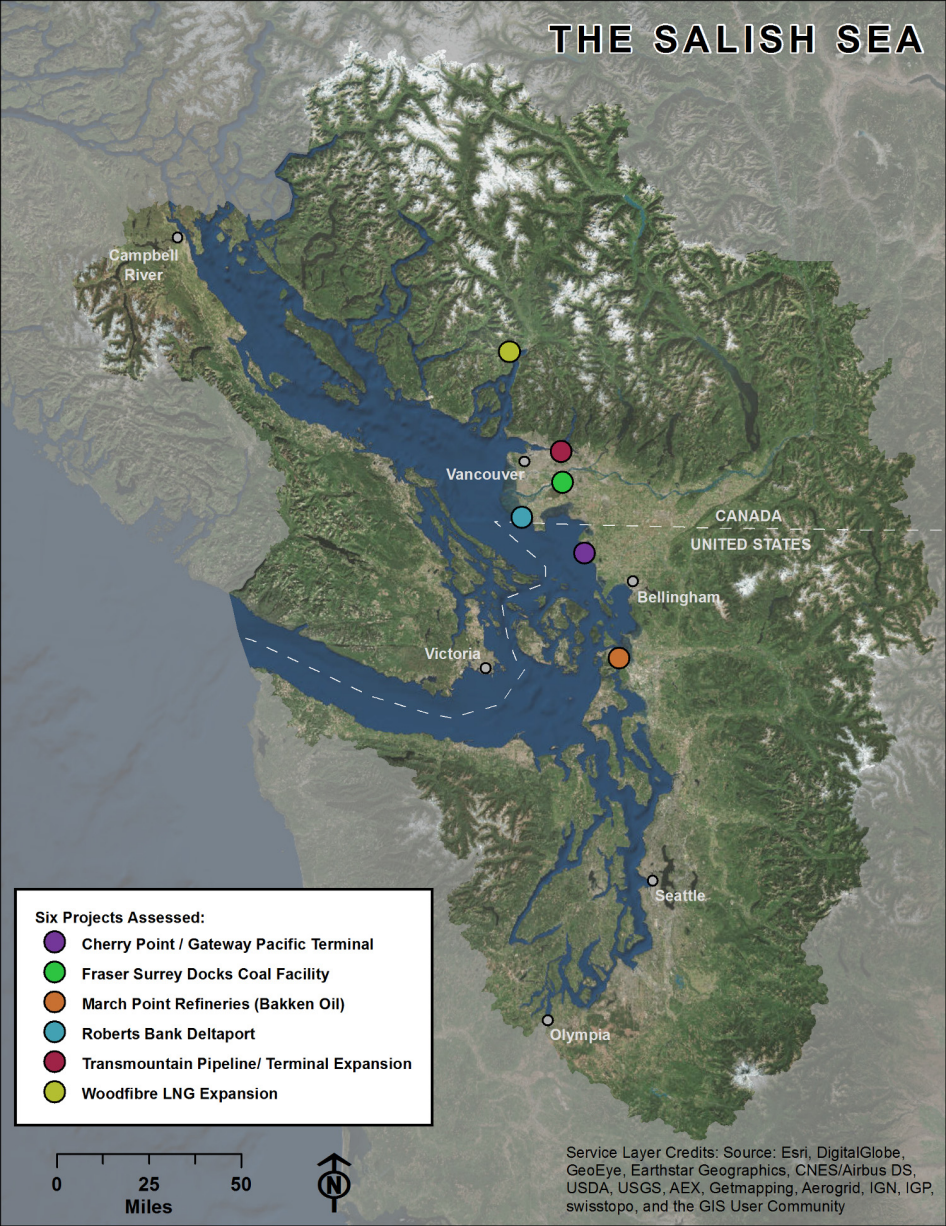
The six projects assessed are located on both sides of the Canadian / United States border, which bisects the Salish Sea and its watershed.

We identified 5 energy-related port development projects and one alteration in transportation (increase in crude oil shipment to existing regional refineries by rail) within the Salish Sea that will significantly increase marine vessel traffic (Table 2). Four are located in British Columbia (Canada) and two across the border in Washington State (USA; Fig 1).

**Table 2:** Energy-related development projects that will increase marine vessel traffic in the Salish Sea.

Project	Location	Product Shipped	Status	Increase in vessel number / year	Shoreline / Marine Development	Environmental Assessment?	Citation
Fraser Surrey Docks Direct Transfer Coal Facility	Surrey / Texada Island, BC	Coal	Approved	454 single formation coal barge tows; undetermined # from Texada Island out the Strait of Juan de Fuca	Yes	Completed	[13–15]
Gateway Pacific Terminal	Whatcom County, WA	Coal and other commodities	Proposed	487 vessels / year (144 Panamax and 77 Capesize)	Yes	Underway	[16]
Rail shipment of Bakken shale oil	Washington Oil Refineries	Crude Oil	In review	Unknown	In some locations	Not needed	[17]
Roberts Bank / Deltaport Terminal 2 Project	Delta, BC	Containers	Proposed	grow from 1.54 million TEU to 2.4–3 million twenty-foot equivalent units (TEUs; # vessels depends on vessel size)	Yes	Underway	[18,19]
Transmountain Pipeline Expansion and Westridge Marine Terminal Expansion	Burnaby, BC	Crude Oil	Proposed	348 tankers / year	Yes	Underway	[20]
Woodfibre Liquefied Natural Gas Terminal	Squamish, BC	Liquefied Natural Gas	Proposed	40 annually (size unknown; likely membrane LNG carriers); Pers. Comm.	Yes	Underway	[21]

Specific details for each project follow:

#### Fraser Surrey Docks Direct Transfer Coal Facility

This approved project will expand a multipurpose marine terminal on the Fraser River (Surrey, BC) by adding a facility that will receive up to four million metric tons (and eventually up to 8 million metric tons in 4–5 years) of coal a year and directly transfer it from rail cars to marine barges [13,15]. Subbituminous coal (intermediate coal between lignite and bituminous coal) from Wyoming or Montana (USA) will then be towed by tug and barge down the Fraser River and north to Texada Island in the Strait of Georgia where it will be stored and eventually loaded onto deep-sea vessels for international export.

#### Gateway Pacific Terminal

This is a proposed multimodal, deep-water terminal (Whatcom County, WA) that would provide storage and handling for the export (and import) of up to 54 million metric tons per year of dry bulk commodities, specifically, calcined petroleum coke, potash, low-sulfur, low-ash coal, and other coal products brought in by rail. The type and quantity of dry bulk commodities could change over time. The proposed terminal would be approximately 334 acres within a total project area of approximately 1,200 acres [16].

#### Increased rail shipment of bakken shale crude oil

Exact numbers could not be specified because this already on-going alteration in transportation does not require an environmental review. Nonetheless, it is projected that shale oil produced from the Bakken fields in North Dakota and Montana will increasingly be shipped by rail to oil refinery facilities in Washington State [17,22]. Recipient unloading and refining facilities in Washington’s portion of the Salish Sea include facilities at Anacortes (Shell and Tesoro), Cherry Point (BP), Ferndale (Phillips 66), and Tacoma (US Oil and Refining), all of which are facilities located on or adjacent to Indian Reservations. As the volume of crude oil coming in for refinement is not known at this time, associated marine vessel traffic increases also are unknown. Increased transportation of crude oil does not require environmental reviews, however construction of new or expanded facilities would. At some facilities, infrastructure development will be necessary to accommodate the increased rail shipments. For example, the Shell facility in Anacortes (WA) submitted an application to construct and operate a crude rail unloading facility (Crude by Rail East Gate Project) that would include four rail unloading stations with the capacity to unload 102 railcars per day [23]. The Tesoro facility is in the process of constructing a new rail unloading system capable of handling four 110-car trains simultaneously, with the intent of receiving up to 50,000 barrels of Bakken shale crude oil a day [24].

#### Roberts Bank Deltaport Terminal 2 Project

This project would build a new three-berth marine container terminal located at Roberts Bank, (Delta, BC) in order to increase shipping container capacity by an additional 2.4 million twenty-foot container equivalent units (TEUs) annually. The project includes a rail tie-in of a lead track to the BCR rail network occupying approximately 1 ha of terrestrial land and will develop the terminal in the intertidal and subtidal area of the Fraser River estuary and delta adjacent to the Roberts Bank Wildlife Management Area, which was established to conserve critical, internationally significant habitat for year-round migrating and wintering waterfowl populations, along with important fish and marine mammal habitat and critical habitat for shorebirds and raptors [19].

#### Trans Mountain Pipeline Expansion and Westridge Marine Terminal Expansion

In order to provide additional transportation capacity for crude oil from Alberta to markets in the Pacific Rim, this project proposes to install new pipeline segments and reactivate existing lines, construct new pump stations, expand existing terminals by adding new tanks and other infrastructure, and construct a new dock complex at Westridge Marine Terminal, Burnaby, BC; [20]. The crude oil would be loaded onto tankers at terminals.

#### Woodfibre Liquefied Natural Gas Terminal

This proposal is to construct a liquefied Natural gas (LNG) production, storage and marine carrier transfer facility on the northwestern shoreline of Howe Sound (near Squamish, BC) for international export of approximately 2.1 million metric tons of LNG annually. Western Canada market hubs will supply LNG to the facility by expanding the existing gas transmission system by FortisBC [21].

### Culturally Important Species

While recognizing that Coast Salish traditions identify all species as important and connected, 50 species were chosen because they are heavily depended upon by Coast Salish for harvest revenue or as a stable food source, particularly culturally or spiritually significant, or are historically part of Coast Salish lifeways (Table 3). Of these species with major cultural importance, 2 were mammals (5% of the 38 species using the ecosystem [25]), 24 were birds (14% of the 172 species using the ecosystem [25]), 8 were fish (3% of the 253 species in the ecosystem [26]), and 10 were invertebrates (0.3% of 3,000 or more macro-invertebrate species estimated to inhabit the Salish Sea [27]). Additionally one was a plant (eelgrass, *Zostera marina*) and 5 were algae species (Table 4). Of the 50 species, 14 species, ecologically distinct units, or distinct population segments of species (28%) are listed by one or more of the four listing jurisdictions in the Salish Sea as endangered, threatened, sensitive, of special concern, or candidates for listing [27].

**Table 3:** Species of major importance for the Coast Salish with provincial, state or Federal listing status [24].

Taxa	Common Name	Latin Name	BC Listing	Washington Listing	Canadian Federal Government Listing	U.S. Endangered Species Act Listing
**Mammal**	Humpback whale	*Megaptera novaengliae*	Blue List	Endangered	Special Concern (COSEWIC); Special Concern (SARA)	Endangered
	Killer whale	*Orcinus orca*	Red List (Southern Residents, Transients and Offshore)	Endangered (Southern Residents, Transients and Offshore)	Endangered (COSEWIC and SARA; (Southern Residents, Transients and Offshore))	Endangered (Southern Residents)
**Avian**	Bald Eagle	*Haliaeetus leucocephalus*	Blue List	NL	NL	NL
	Great Blue Heron	*Ardea herodias*	NL	NL	NL	NL
	Double-crested Cormorant	*Phalacrocorax auritus*	Red List	Candidate	NL	NL
	Common Murre	*Uria aalge*	Blue List	Candidate	Candidate (COSEWIC)	Species of Concern to Not Listed
	Cassin’s Auklet	*Ptychoramphus aleuticus*	NL	NL	NL	NL
	Sooty Shearwater	*Puffinus griseus*	NL	Sensitive	NL	Species of Concern
	Ring-necked Duck	*Aythya collaris*	NL	NL	NL	NL
	Tufted Duck	*Aythya fuligula*	NL	NL	NL	NL
	King Eider	*Somateria spectabilis*	NL	NL	NL	NL
	Common Merganser	*Mergus merganser*	NL	NL	NL	NL
	Common Goldeneye	*Bucephala clangula*	NL	NL	NL	NL
	Barrow’s Goldeneye	*Bucephala islandica*	NL	NL	NL	NL
	Hooded Merganser	*Lophodytes cucullatus*	NL	NL	NL	NL
	Red-breasted Merganser	*Mergus serrator*	NL	NL	NL	NL
	Long-tailed Duck	*Clangula hyemalis*	Blue List	NL	NL	NL
	Harlequin Duck	*Histrionicus histrionicus*	NL	NL	NL	NL
	White-winged Scoter	*Melanitta fusca*	NL	NL	NL	NL
	Black Scoter	*Melanitta nigra*	NL	NL	NL	NL
	Surf Scoter	*Melanitta perspicillata*	Blue List	NL	NL	NL
	Yellow-billed Loon	*Gavia adamsii*	Blue List	NL	Candidate (COSEWIC)	Candidate
	Arctic Loon	*Gavia arctica*	NL	NL	NL	NL
	Common Loon	*Gavia immer*	NL	Sensitive	NL	NL
	Pacific Loon	*Gavia pacifica*	NL	NL	NL	NL
	Red-throated Loon	*Gavia stellata*	NL	NL	NL	NL
**Fish**	Pink Salmon	*Oncorhynchus gorbuscha*	NL	NL	NL	NL
	Chum Salmon	*Oncorhynchus keta*	NL	NL	NL	NL
	Coho Salmon	*Oncorhynchus kisutch*	NL	NL	NL	NL
	Steelhead	*Oncorhynchus mykiss*	NL	NL	NL	NL
	Sockeye Salmon	*Oncorhynchus nerka*	NL	NL	NL	NL
	Chinook Salmon	*Oncorhynchus tshawytscha*	NL	Candidate (Puget Sound)	Endangered (COSEWIC, Fraser River)	Threatened (Puget Sound)
	Eulachon	*Thaleichthys pacificus*	Candidate	Endangered (Central Pacific Coast & Frasier River; COSEWIC)	Threatened (Southern)	NL
	Pacific Herring	*Clupea pallasii*	NL	NL	NL	NL
**Invertebrate**	Dungeness crab	*Metacarcinus magister*	NL	NL	NL	NL
	Spot prawn	*Pandalus platyceros*	NL	NL	NL	NL
	Olympia oyster	*Ostrea conchaphila*	Blue List	Candidate	Special Concern (COSEWIC and SARA)	NL
	Butter clams	*Saxidomus gigantea*	NL	NL	NL	NL
	Native littleneck clams	*Prototheca abrupta*	NL	NL	NL	NL
	Geoduck clam	*Panopea generosa*	NL	NL	NL	NL
	Northern abalone	*Haliotis kamstchatkana*	Red List	Candidate	Endangered (COSEWIC); Threatened to Endangered (SARA)	Species of Concern
	Blue mussel	*Mytilus edulus*	NL	NL	NL	NL
	Red urchin	*Strongylocentrotus franciscanus*	NL	NL	NL	NL
	California sea cucumber	*Parastichopus californicus*	NL	NL	NL	NL
**Plant or Algae**	Eelgrass	*Zostera marina*	NL	NL	NL	NL
	*Fucus*	*Fucus distichus*	NL	NL	NL	NL
	Nori	*Porphyra spp*.	NL	NL	NL	NL
	Bull Kelp	*Nereocystis luetkeana*	NL	NL	NL	NL
	Sea Lettuce	*Ulva lactuca*	NL	NL	NL	NL
	*Aleria*/Wing Kelp	*Aleria marginata*	NL	NL	NL	NL

Note, NL = not listed.

**Table 4:** Rankings for project pressure/species interaction (likely, possibly, unlikely, data deficient) for all possible project components.

		Master Impact (all potential project components included)							
		Increased Vessel Traffic			Localized Impacts				
Taxa	Species	Spill	Underwater Noise	Vessel Strike	Shoreline Development	Harbor Spill	Pipeline Spill	Coal Dust Accumulation	Nearshore LNG Explosion
**Mammal**	Humpback whale	Possibly [28]	Likely [29]	Likely [30]	Possibly [16]	Unlikely [a]	Unlikely [a]	Data Deficient [31]	Unlikely [a]
	Killer whale	Likely [32]	Likely [33]	Unlikely [b]	Possibly [16]	Unlikely [c]	Unlikely [c]	Data Deficient [31]	Possibly [34]
**Avian**	Bald Eagle	Likely [33]	Unlikely [d]	Unlikely [d]	Possibly [16]	Likely [35]	Likely [35]	Data Deficient [31]	Possibly [34]
	Great Blue Heron	Possibly [36]	Unlikely [d]	Unlikely [d]	Possibly [16]	Possibly [36]	Possibly [36]	Data Deficient [31]	Possibly [34]
	Double-crested Cormorant	Likely [37]	Unlikely [d]	Unlikely [d]	Possibly [16]	Likely [37]	Possibly [37]	Data Deficient [31]	Possibly [34]
	Common Murre	Likely [37]	Unlikely [d]	Unlikely [d]	Unlikely [e]	Possibly [37]	Unlikely [e]	Data Deficient [31]	Unlikely [e]
	Cassin’s Auklet	Likely [37]	Unlikely [d]	Unlikely [d]	Unlikely [e]	Unlikely [e]	Unlikely [e]	Data Deficient [31]	Unlikely [e]
	Sooty Shearwater	Likely [37]	Unlikely [d]	Unlikely [d]	Unlikely [e]	Unlikely [e]	Unlikely [e]	Data Deficient [31]	Unlikely [e]
	Ring-necked Duck	Likely [37]	Unlikely [d]	Unlikely [d]	Possibly [16]	Likely [37]	Possibly [37]	Data Deficient [31]	Possibly [34]
	Tufted Duck	Likely [37]	Unlikely [d]	Unlikely [d]	Unlikely [e]	Likely [37]	Possibly [37]	Data Deficient [31]	Possibly [34]
	King Eider	Likely [37]	Unlikely [d]	Unlikely [d]	Unlikely [e]	Unlikely [e]	Unlikely [e]	Data Deficient [31]	Unlikely [e]
	Common Merganser	Likely [37]	Unlikely [d]	Unlikely [d]	Unlikely [e]	Likely [37]	Possibly [37]	Data Deficient [31]	Possibly [34]
	Common Goldeneye	Likely [37]	Unlikely [d]	Unlikely [d]	Possibly [16]	Likely [37]	Possibly [37]	Data Deficient [31]	Possibly [34]
	Barrow’s Goldeneye	Likely [37]	Unlikely [d]	Unlikely [d]	Possibly [16]	Likely [37]	Possibly [37]	Data Deficient [31]	Possibly [34]
	Hooded Merganser	Likely [37]	Unlikely [d]	Unlikely [d]	Possibly [16]	Likely [37]	Possibly [37]	Data Deficient [31]	Possibly [34]
	Red-breasted Merganser	Likely [37]	Unlikely [d]	Unlikely [d]	Possibly [16]	Likely [37]	Possibly [37]	Data Deficient [31]	Possibly [34]
	Long-tailed Duck	Likely [37]	Unlikely [d]	Unlikely [d]	Unlikely [e]	Likely [37]	Possibly [37]	Data Deficient [31]	Possibly [34]
	Harlequin Duck	Likely [37]	Unlikely [d]	Unlikely [d]	Possibly [16]	Likely [37]	Possibly [37]	Data Deficient [31]	Possibly [34]
	White-winged Scoter	Likely [37]	Unlikely [d]	Unlikely [d]	Unlikely [e]	Likely [37]	Possibly [37]	Data Deficient [31]	Possibly [34]
	Black Scoter	Likely [37]	Unlikely [d]	Unlikely [d]	Unlikely [e]	Likely [37]	Possibly [37]	Data Deficient [31]	Possibly [34]
	Surf Scoter	Likely [37]	Unlikely [d]	Unlikely [d]	Unlikely [e]	Likely [37]	Possibly [37]	Data Deficient [31]	Possibly [34]
	Yellow-billed Loon	Likely [37]	Unlikely [d]	Unlikely [d]	Unlikely [e]	Likely [37]	Possibly [37]	Data Deficient [31]	Unlikely [e]
	Arctic Loon	Likely [37]	Unlikely [d]	Unlikely [d]	Unlikely [e]	Likely [37]	Possibly [37]	Data Deficient [31]	Unlikely [e]
	Common Loon	Likely [37]	Unlikely [d]	Unlikely [d]	Possibly [16]	Likely [37]	Possibly [37]	Data Deficient [31]	Possibly [34]
	Pacific Loon	Likely [37]	Unlikely [d]	Unlikely [d]	Unlikely [e]	Likely [37]	Likely [37]	Data Deficient [31]	Unlikely [e]
	Red-throated Loon	Likely [37]	Unlikely [d]	Unlikely [d]	Unlikely [e]	Likely [37]	Possibly [37]	Data Deficient [31]	Unlikely [e]
**Fish**	Pink Salmon	Likely [38]	Possibly [39–41]	Unlikely [f]	Possibly [16]	Likely [38]	Likely [38]	Data Deficient [31]	Possibly [34]
	Chum Salmon	Possibly [42]	Possibly [39–41]	Unlikely [f]	Possibly [16]	Possibly [42]	Possibly [42]	Data Deficient [31]	Possibly [34]
	Coho Salmon	Likely [43]	Possibly [39–41]	Unlikely [f]	Possibly [16]	Likely [43]	Likely [43]	Data Deficient [31]	Possibly [34]
	Steelhead	Possibly [42]	Possibly [39–41]	Unlikely [f]	Possibly [16]	Possibly [42]	Possibly [42]	Data Deficient [31]	Possibly [34]
	Sockeye Salmon	Likely [43]	Possibly [39–41]	Unlikely [f]	Possibly [16]	Likely [43]	Likely [43]	Data Deficient [31]	Possibly [34]
	Chinook Salmon	Likely [42]	Possibly [39–41]	Unlikely [f]	Possibly [16]	Likely [42]	Likely [42]	Data Deficient [31]	Possibly [34]
	Eulachon	Possibly [44,45]	Possibly [39–41]	Unlikely [f]	Unlikely [g]	Possibly [44]	Possibly [44]	Data Deficient [31]	Possibly [34]
	Pacific Herring	Likely [44,45]	Possibly [39–41]	Unlikely [f]	Likely [16]	Likely [44]	Likely [44]	Data Deficient [31]	Possibly [34]
**Invertebrate**	Dungeness crab	Unlikely [46]	Unlikely [h]	Unlikely [h]	Unlikely [i]	Unlikely [46]	Unlikely [46]	Data Deficient [31]	Possibly [34]
	Spot prawn	Possibly [47]	Unlikely [h]	Unlikely [h]	Unlikely	Unlikely	Unlikely	Data Deficient [31]	Possibly [34]
	Olympia oyster	Likely [48]	Unlikely [h]	Unlikely [h]	Unlikely	Likely [48]	Possibly [48]	Data Deficient [31]	Possibly [34]
	Butter clam	Likely [48]	Unlikely [h]	Unlikely [h]	Unlikely	Likely [48]	Possibly [48]	Data Deficient [31]	Possibly [34]
	Native littleneck clam	Likely [48]	Unlikely [h]	Unlikely [h]	Unlikely	Likely [48]	Possibly [48]	Data Deficient [31]	Possibly [34]
	Geoduck clam	Likely [48]	Unlikely [h]	Unlikely [h]	Unlikely	Unlikely	Unlikely	Data Deficient [31]	Possibly [34]
	Northern abalone	Unlikely	Unlikely [h]	Unlikely [h]	Unlikely	Unlikely	Unlikely	Data Deficient [31]	Unlikely
	Blue mussel	Likely [48]	Unlikely [h]	Unlikely [h]	Unlikely	Likely [48]	Possibly [48]	Data Deficient [31]	Possibly [34]
	Red urchin	Unlikely [46]	Unlikely [h]	Unlikely [h]	Unlikely	Unlikely [46]	Unlikely [46]	Data Deficient [31]	Possibly [34]
	California sea cucumber	Unlikely [46]	Unlikely [h]	Unlikely [h]	Unlikely	Unlikely [46]	Unlikely [46]	Data Deficient [31]	Possibly [34]
**Plant or Algae**	Eelgrass	Unlikely [49]	Unlikely [h]	Unlikely [h]	Likely [50]	Unlikely [49]	Unlikely [49]	Data Deficient [31]	Possibly [34]
	*Fucus*	Likely [51]	Unlikely [h]	Unlikely [h]	Unlikely	Likely [51]	Possibly [51]	Data Deficient [31]	Possibly [34]
	Nori	Possibly [52]	Unlikely [h]	Unlikely [h]	Unlikely	Possibly [52]	Possibly [52]	Data Deficient [31]	Possibly [34]
	Bull Kelp	Likely [53]	Unlikely [h]	Unlikely [h]	Unlikely	Likely [53]	Possibly [53]	Data Deficient [31]	Possibly [34]
	Sea Lettuce	Possibly [52]	Unlikely [h]	Unlikely [h]	Unlikely	Possibly [52]	Possibly [52]	Data Deficient [31]	Possibly [34]
	*Aleria*/Wing Kelp	Possibly [52]	Unlikely [h]	Unlikely [h]	Unlikely	Possibly [52]	Possibly [52]	Data Deficient [31]	Possibly [34]

Notes

^a^ Species unlikely to occur in specific localized habitat (Calambokidis J, Steiger G, Ellifrit D, Troutman B, Bowlby E. Distribution and abundance of humpback whales (*Megaptera novaeangliae*)and other marine mammals off the northern Washington coast. Fish Bull. 2004; 102:563–580.)

^b^ Vessel strike rarely documented as mortality factor for species (Jensen AS, Silber GK. Large Whale Ship Strike Database. U.S. Department of Commerce, NOAA Tech Memo 2003; NMFS-OPR 37 pp.)

^c^ Species unlikely to occur in specific localized habitat (National Marine Fisheries Service. 2008. Recovery Plan for Southern Resident Killer Whales (*Orcinus orca*). National Marine Fisheries Service, Northwest Region, Seattle, Washington.)

^d^ Underwater noise and vessel strike not believed to be a threat to marine birds (Vilchis IL, Kreuder Johnson C, Evenson JR, Pearson SF, Barry K, Davidson P, Raphael M, Gaydos JK. Assessing ecological correlates of marine bird declines to inform marine conservation. Conservation Biology. 2014. DOI: 10.1111/cobi.12378.

^e^ Species unlikely to occur in specific localized habitat (Wahl TR, Tweit B, Mlodinow SG. Birds of Washington. Oregon State University Press, Corvallis, Oregon; 2005.).

^f^ Vessel strike not considered a threat to marine fish species.

^g^ Species unlikely to occur in specific localized habitat (Pietsch TW, Orr JW. Fishes of the Salish Sea: A Compilation and Distributional Analysis. NOAA Prof Paper NMFS 18, U.S. Dept Comm. 2015. pp 106.

^h^ Underwater noise and vessel strike not considered a threat to marine invertebrate species.

^i^ Species unlikely to occur in specific localized habitat (Encyclopedia of Life www.eol.org).

### Impacts and Data Gaps

Each project had 8 potential impacts (pressures; Table 4). All six projects had the 3 potential impacts associated with increased marine vessel traffic: oil spill, vessel noise and vessel strike. Projects varied in their potential for localized impacts including shoreline development, harbor oil spill, pipeline spill, coal dust accumulation and nearshore LNG explosion. Potential impacts by project are detailed below.

#### Fraser Surrey Docks Direct Transfer Coal Facility

In addition to marine vessel traffic pressures, the Fraser Surrey Docks Direct Transfer Facility included 3 of 5 potential localized impacts: shoreline development, harbor spill and coal dust. Each of the 6 pressures had the potential to impact each of the 50 species for 300 potential pressure/species interactions (Table 5). Of those, 70 (23%) were likely to impact species, 45 (15%) could possibly have impact, and 134 (45%) were unlikely to have impact. The remaining 16.7% (n = 50) were data deficient, precluding assessment.

**Table 5:** Number of pressure/species interactions by project with breakdown on potential for negative impact to be likely, possible, unlikely, or unknown (data deficient).

		Interaction Potential to have impact			
Project	Pressure / Species interactions	Likely	Possibly	Unlikely	Data Deficient
Fraser Surrey Docks Direct Transfer Coal Facility	300	23%	15%	45%	17%
Gateway Pacific Terminal	300	23%	15%	45%	17%
Rail shipment of Bakken shale oil	250	28%	18%	54%	0%
Roberts Bank Deltaport Terminal 2 Project	250	28%	18%	54%	0%
Trans Mountain Pipeline Expansion and Westridge Marine Terminal Expansion	300	25%	25%	50%	0%
Woodfibre Liquified Natural Gas Terminal	300	23%	28%	49%	0%

#### Gateway Pacific Terminal

The Gateway Pacific Terminal had the same 6 potential impacts (pressures) as the Fraser Surrey Docks Direct Transfer Coal Facility and consequently had the same rankings for the 300 potential pressure species interactions: 70 likely impacts, 45 possible impacts, 134 unlikely impacts and 50 that were data deficient.

#### Increased rail shipment of Bakken shale crude oil

Increasing rail shipment of crude oil had all 3 pressures associated with increased marine vessel traffic and 2 potential localized impacts (shoreline development and harbor spill), making 250 potential pressure/species interactions. Of those, 71 (28%) were likely to impact, 44 (18%) could possibly impact, and 135 (54%) were unlikely to cause impact.

#### Roberts Bank Deltaport Terminal 2 project

In addition to all 3 pressures associated with increased marine vessel traffic, this project had localized pressures of shoreline development and harbor spill for 250 potential pressure/species interactions. Impact was likely for 70 (28%), possibly present for 44 (18%) and unlikely for 136 (54%).

#### Trans Mountain Pipeline Expansion and Westridge Marine Terminal Expansion

This project had the 3 increased marine vessel traffic-associated pressures as well as 3 localized ones: shoreline development, harbor spill and pipeline spill. Of the 300 potential pressure/species interactions, 76(25%) were likely to impact, 75(25%) could possibly impact, and 149 (50%) were unlikely to have impact on species.

#### Woodfibre Liquefied Natural Gas Terminal

Development of this proposed liquefied Natural gas production, storage and marine carrier transfer facility had the 3 pressures associated with increased marine vessel traffic and the 3 localized impacts of shoreline development, harbor spill or nearshore LNG explosion for 300 potential pressure/species interactions. Of those interactions 70 (24%) were likely to have impacts, 83 (28%) could possibly impact, and 146 (49%) were unlikely to have impact.

## Discussion

All 6 projects evaluated have the potential to adversely affect species that are highly important to indigenous Coast Salish people. Likely impact ranged from 23 to 28% of the possible pressure/species scenarios with the possibility to impact species in 15 to 28% additional instances. Cumulatively, these projects also have the potential to additively, synergistically, or antagonistically impact species of major cultural importance [6]. While it is not clear which impacts will be additive, synergistic, or potentially antagonistic, studies that manipulate multiple stressors in marine ecosystems suggest that threats associated with these six projects are likely to have an overall additive or even synergistic interaction [6,54] and therefore impact species of major cultural importance to the Coast Salish, an important concept that would be lost by merely evaluating each project independently.

While mitigation efforts never completely remove risk, efforts have been made to develop mitigation strategies to minimize the potential for increased oil spills for a subset (n = 3) of these projects [55]. Mitigating the potential of increased risk of vessel strike of listed humpback whales (*Megaptera novaeangliae* [30]) or the impact of increased underwater noise on killer whales (*Orcinus orca*[31]), humpback whales [29], or possibly on the 8 species of teleost fish [39–41] could be more challenging. Scientists are just beginning to understand the association with sound scape and habitat quality for marine mammals and fishes in the Salish Sea [56], and the importance of this pressure should not be overlooked or underestimated when evaluating potential impacts of increased marine vessel traffic in the Salish Sea.

Unburnt coal commonly enters the marine environment through a variety of anthropogenic mechanisms. While the direct and indirect physical effects on organisms are similar to other types of suspended and deposited sediments (abrasion, increased water turbidity, reduced photosynthetic performance, clogging of feeding and respiratory organs of some species, egg and larval mortality, etc.), the chemical effects have not been well studied [57]. The lack of data on the potential impact of coal dust on marine organisms prevents a thorough evaluation of risk at this time. It is clear that coal will likely enter the marine ecosystem from new coal loading facilities [31,57]. Data from other parts of the country suggest that coal particulate matter has the potential to transport arsenic into soils, which could impact marine organisms and or potentially contaminate shellfish or finfish [58]. Alternately, coal particles could absorb PAHs and other similar chemicals from the environment similar to activated carbon [59]. The paucity of marine-focused studies on the toxic effects of coal at the organism or the population level argues that more detailed studies are needed [57].

## Conclusions

### Data Gaps

Sufficient data exist to suggest that an oil spill resulting from increased vessel traffic would impact or potentially impact 45 of 50 important species and consequently greatly impact the Coast Salish. Data are not as robust for other pressures. To help understand the potential impact of underwater noise on nearly all of the 50 species of major cultural importance, data are needed to help assess potential impacts associated with increased marine vessel traffic in the Salish Sea. Similarly, data on the potential toxic impacts of coal on all 50 species would enable more intelligent estimates for risks associated with spilled coal in the ecosystem.

While the health of populations of some of the identified species populations have been well studied, many have not, and risk assessment will require more extensive evaluation of the current state of health for these understudied species. It cannot be assumed that the identified species are currently robust and healthy, and not subject to multiple other pressures that increase their vulnerability to impact from additional stressors such as increased vessel traffic. While this is beyond the scope of the report, the fact that 28% of these species also are listed by one or more governmental jurisdiction within the region as endangered, threatened, sensitive, of special concern, or as candidates for listing, suggests that for a substantial portion of these culturally important species, populations are not in a resilient state and might not easily cope with increased stressors.

### Decision Making

While not all data are equally important in decision-making processes, the collection of relevant data is needed to move from assessment to decision-making [60]. In addition to identifying and researching the priority data gaps, work needs to be completed estimating the probability of risk and the uncertainty associated with each pressure/species interaction. Findings can then be taken back to the Coast Salish to determine significance of identified risks. Ultimately, an established process such as structured decision making [60] should be used to better understand how Coast Salish health and wellbeing would be impacted by these development projects.

### Management needs to establish a mechanism for addressing transboundary issues

Proposed or on-going projects that would increase marine vessel traffic in the Salish Sea exist on the US and on the Canadian sides of the Salish Sea ecosystem. The Salish Sea is not unique and most multinational ecosystems routinely experience multiple potential risks that occur independently within multiple jurisdictions. Despite the fact that risk assessments will only be accurate when considered concurrently with other potential and ongoing development, such cumulative assessments are often not conducted if formal mechanisms to support transboundary evaluation do not exist. While the indigenous Coast Salish people recognize this need and are working to address it, transboundary ecosystems such as the Salish Sea are left vulnerable to many cumulative pressures due to the absence of established collaborative decision-making processes. The people of the Salish Sea and other multinational ecosystems need to develop structured mechanisms for dealing with such issues. Within the Salish Sea, a government-sponsored process such as a US—Canadian International Joint Commission (IJC; www.ijc.org) might be suitable to deal with United States / Canadian transboundary problems. The IJC is designed to help Canada and the United States prevent disputes over transboundary waters. Alternately, a novel non-governmental Salish Sea commission could be created that represents the Coast Salish and non-Native people on both sides of the border as well as US and Canadian State, Provincial, and Federal governing bodies and management agencies.

### Consequences of failing at transboundary ecosystem management

The health and welfare of Coast Salish Tribes and First Nations are inextricably linked to the wellbeing of the natural environment. The identified six major development projects occurring in one ecosystem that is shared by two different countries could individually and cumulatively affect species that are of major importance to the Coast Salish. Ultimately these projects could likely negatively affect Coast Salish lifeways at a time when Coast Salish tribal treaty rights are already at risk [61].

As an ecosystem, the Salish Sea functions without regard to international borders or myriad governing agencies [4]. This ecosystem's complex web of political and management oversight, however, is the only option for mitigating anthropogenic impacts on the ecosystem. Nonetheless, there is no governing body that demands all six projects be evaluated for their cumulative impact. This is a failure in coastal ecosystem management that stands to have direct impact on the Coast Salish and likely on most of the 7 million other people that also depend on this ecosystem. An over-arching body that represents the numerous managers and stakeholders and works to collaboratively govern the Salish Sea is needed.

On a global scale, this preliminary evaluation of the threats from multiple energy-related development projects in the Salish Sea shows the value of evaluating impacts on the ecosystem-scale and highlights the serious need for managers of multinational ecosystems to actively collaborate and evaluate threats on the ecosystem scale. Following that is the need for future risk assessment to be done on the scale of the ecosystem as well. The case of the Salish Sea is not merely an anomaly, but is exemplary of many ecosystems around the world that are under multiple jurisdictions and in jeopardy. Establishing a transnational authority to evaluate cumulative risk for the Salish Sea would not only benefit this ecosystem and its constituents, it would serve as a model for other multinational marine ecosystems working to evaluation threats in the face of continued resource development.
